# Higher prevalence of gestational diabetes in euthyroid women with thyroid autoimmunity who were expecting a female fetus

**DOI:** 10.1530/ETJ-24-0339

**Published:** 2025-02-17

**Authors:** Madhu Prasai, Manon Lomré, Emna Jelloul, Pierre Kleynen, Flora Veltri, Georgiana Sitoris, Lidia Grabczan, Serge Rozenberg, Kris G Poppe

**Affiliations:** ^1^ Department of Endocrinology, Centre Hospitalier Universitaire (CHU) Saint Pierre, Université Libre de Bruxelles (ULB), Brussels, Belgium; ^2^ Departement of Gynaecology and Obstetrics, Centre Hospitalier Universitaire (CHU) Saint Pierre, Université Libre de Bruxelles (ULB), Brussels, Belgium

**Keywords:** fetal gender, gestational diabetes, pregnancy, thyroid autoimmunity

## Abstract

**Objective:**

In the general population, women pregnant with a male fetus (MF) have a higher prevalence of gestational diabetes mellitus (GDM) compared with those pregnant with a female fetus (FF). Some studies suggest a higher prevalence of GDM in euthyroid pregnant women with thyroid autoimmunity (TAI+) compared with women without TAI (TAI−). However, whether the impact of TAI on GDM correlates with fetal gender has not been documented.

**Design/methods:**

A single-center cohort study including 1201 women who were screened at a median of 12 (11–14) weeks of pregnancy for thyroid disorders (TSH, free T4 and thyroid peroxidase antibodies (TPOAb)) and at 24–28 weeks for GDM with an oral glucose tolerance test. Exclusion criteria were pre-pregnancy diabetes or hypertension, thyroid dysfunction (treated or untreated) before and after screening, thyroid screening after 20 weeks of pregnancy and assisted pregnancies. The diagnosis of GDM was based on the 2013 WHO criteria, and that of TAI by increased TPOAb levels (≥60 kIU/L).

**Results:**

Overall, 622 women were expecting a FF (51.8%) and 579 a MF (48.2%). Seventy-five women were TAI+ (6.2%). The overall prevalence of GDM was 19.6%, 28% in TAI+ women and 19% in TAI− women (*P* = 0.008 after adjustment for confounders). In women who were expecting a FF, the prevalence of GDM was 34.4% in TAI+ women vs 19.2% in TAI− women; *P* = 0.002.

**Conclusions:**

The prevalence of GDM was increased in euthyroid TAI+ women, but only in the case of pregnancies with a FF. This is opposite to the result observed in the general population and deserves more research to explore the underlying mechanisms.

## Introduction

Gestational diabetes mellitus (GDM) is a common condition and is associated with complications such as preeclampsia and macrosomia (1). Several factors have been associated with a higher prevalence of GDM, such as maternal age, obesity, history of GDM and ethnic background (2). In some studies, women pregnant with a male fetus (MF) had a higher prevalence of GDM compared with women pregnant with a female fetus (FF), suggesting that male fetal gender may be an additional risk factor for the development of GDM (3, 4, 5). The precise underlying mechanism for this association remains unclear, but some of the effect may be mediated by different levels in human placenta lactogen, involved in β-cell compensation during pregnancy (6). Other pathologies often found during pregnancy are subclinical hypothyroidism (SCH) with or without thyroid autoimmunity (TAI). Both SCH and TAI have been associated with a higher risk of GDM via different pathways, including increased insulin resistance (IR) (2, 7, 8). No data have been published regarding the prevalence of GDM in euthyroid women with TAI and in relation to fetal gender.

The aim of this study was to investigate whether the prevalence of GDM in euthyroid women with TAI (TAI+) was different compared with that in women without TAI (TAI−) and to stratify the results according to fetal gender.

## Materials and methods

### Study design and definitions

This cohort study took place in the department of Endocrinology and Gynecology/Obstetrics in a public university hospital, CHU Saint Pierre in Brussels, Belgium. Eligibility criteria comprised all women with ongoing pregnancies who performed their laboratory screening and full obstetric follow-up in our center during the period of January 2013 to December 2014.

Exclusion criteria for this study were multiple pregnancy or by assisted reproductive technology (ART), known diabetes mellitus, first trimester fasting glucose ≥92 mg/dL, treatment with levothyroxine (LT4) or antithyroid medication either before screening or subsequently during pregnancy, overt hyperthyroidism and overt or SCH.

**Figure 1:**
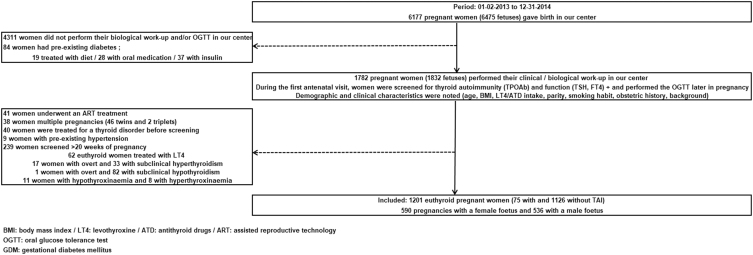
Flowchart of the study selection process.

Figure 1 details the study selection process.

During the first antenatal consultation, demographic parameters and obstetric data were collated, along with routine blood tests, including serum thyrotropin (TSH), FT4, thyroid peroxidase antibodies (TPOAb) and fasting glucose. An oral glucose tolerance test (OGTT) was performed at 24–28 weeks of pregnancy.

In a previous study, we determined institutional-specific first-trimester reference ranges for serum TSH and FT4, according to the ATA-GL recommendations (9). The reference range for serum TSH (2.5–97.5th percentile) was 0.06–3.74 mIU/L and 10.29–18.02 pmol/L for serum FT4 (10). TAI was defined as TPOAb levels ≥60 kIU/L. For this study, two groups were established based on the presence of TAI (TAI+, *n* = 75) and absence of it (TAI−, *n* = 1126).

Gestational age was based on ultrasound findings and expressed in full weeks and days of amenorrhea. Smoking was stratified as yes/no (women who stopped smoking during pregnancy were also considered as smokers).

GDM was diagnosed by a 75 g OGTT performed between 24 and 28 weeks of pregnancy according to WHO criteria: fasting glucose ≥92 mg/dL or 1-h postprandial glucose ≥180 mg/dL or 2 h ≥153 mg/dL (11).

The study was approved by the ethical review board (AK/15-11-114/4568). The need for written consent from study participants was waived due to the retrospective analysis of routinely collected data.

### Serum assays

All blood test analyses were performed by the laboratory of CHU Saint Pierre.

Serum TSH, FT4 and TPOAb levels were measured using the Chemiluminescence Centaur XP Siemens immunoanalyzer. Nonpregnant reference values were 0.3–4.0 mIU/L, 10.3–25.7 pmol/L (0.8–2.0 ng/dL) and <60 kIU/L for TSH, FT4 and TPOAb, respectively. Total imprecision CVs were 6.9, 4.2 and 7.6% for TSH, FT4 and TPOAb, respectively. Plasma glucose was measured by an automated colorimetric-enzymatic method on a Hitachi/Roche Modular P analyzer; CV was 1%.

### Statistical analysis

Data were stored in a Microsoft Excel database and statistical analyses performed using the StatPlus:mac, Analyst Soft Inc. – a statistical analysis program for macOS; version v8 (https://www.analystsoft.com/en/). Descriptive statistics are presented as the mean ± standard deviation (SD) for normally distributed and median (interquartile range (IQR)) for skewed variables. Comparison between groups was performed by Chi^2^ or Fisher’s exact tests (according to the number of events) for categorical data and by Student’s *T*-test or Mann–Whitney U test for continuous data (according to the distribution).

*P* values for the prevalence of GDM are given as crude and adjusted with correction for age (>34 years, body mass index (BMI) ≥30 kg/m^2^) and history of GDM. *P* values were considered significant whenever *P* < 0.017 (0.05/3) for three comparisons.

## Results

Table 1 shows baseline demographic and obstetric parameters in TAI+ and TAI− women and according to the fetal gender.

**Table 1:** Demographic and obstetric parameters in women with and without TAI and according to the fetal gender. Continuous data are expressed as the mean ± SD or as median (IQ 1–3) according to the distribution and categorical data as *n* (%). Statistically significant values are presented in bold.

Demographic and obstetric data	All women	TAI		
		Positive (+)	Negative (−)	*P*-level*
Total *n*	**1201**	**75 (6.2%)**	**1126 (93.8%)**	
FF	**622 (51.8%)**	**32 (5.1%)**	**590 (94.9%)**	
MF	**579 (48.2%)**	**43 (7.4%)**	**536 (92.6%)**	
Maternal age (years)	30 (26–34)	32 (27–36)	30 (26–34)	0.029
FF		29 (26–36)	30 (25–34)	0.502
MF		34 (28–35)	30 (26–34)	0.022
Maternal age ≥35 years	283 (23.6%)	27 (36.0%)	256 (22.7%)	**0.009**
FF	144 (23.2%)	11 (34.4%)	133 (22.5%)	0.122
MF	139 (24.0%)	16 (37.2%)	123 (22.9%)	0.035
BMI pre-pregnancy (kg/m^2^)	25 (22–28)	24 (22–27)	25 (22–28)	**0.013**
FF		23 (21–26)	25 (22–28)	**0.011**
MF		25 (22–27)	25 (22–29)	0.295
Obesity (BMI ≥30 kg/m^2^)	187 (15.6%)	5 (6.7%)	182 (16.2%)	0.028
FF	93 (15.0%)	1 (3.1%)	92 (15.6%)	0.054
MF	94 (16.2%)	4 (5.3%)	90 (16.8%)	0.200
Other than Caucasian background	914 (76.1%)	51 (68.0%)	863 (76.6%)	0.089
FF	472 (75.9%)	20 (62.5%)	452 (76.6%)	0.069
MF	442 (76.3%)	31 (72.1%)	411 (75.7%)	0.499
Multiparity (> = 2)	383 (31.9%)	23 (30.7%)	360 (32%)	0.814
FF	203 (32.6%)	10 (31.3%)	193 (32.7%)	0.864
MF	180 (31.1%)	13 (30.2%)	167 (31.2%)	0.900
History of ≥2 first trimester MC	85 (7.1%)	5 (6.7%)	80 (7.1%)	0.886
FF		1 (3.1%)	38 (6.4%)	0.451
MF		4 (9.3%)	42 (7.8%)	0.733
Smoking during pregnancy	173 (14.4%)	17 (22.7%)	156 (13.9%)	0.035
FF		7 (21.9%)	84 (14.2%)	0.234
MF		10 (23.3%)	72 (13.4%)	0.075

BMI, body mass index; MC, miscarriage; FF, female fetus; MF, male fetus; TAI, thyroid autoimmunity; SD, standard deviation.

* TAI+ all vs TAI− all/TAI+ FF vs TAI− FF/TAI+ MF vs TAI− MF; *P* values were considered significant whenever *P* < 0.017 (0.05/3) for three comparisons.

The median (IQR) age was 30 (26–34) years, and was higher in TAI+ vs TAI− women (32 (27–36) vs 30 (26–34) years; *P* = 0.029) and in TAI+ women pregnant with a MF vs a FF (34 (28–35) vs 30 (26–34) years; *P* = 0.022). More TAI+ women were aged ≥35 years than TAI− women (36.0 vs 22.7%; *P* = 0.009).

BMI was 25 (22–28) kg/m^2^ for all women, and was lower in TAI+ vs TAI− women (24 (22–27) vs 25 (22–28) kg/m^2^; *P* = 0.013) and in TAI+ women pregnant with a FF vs a MF (23 (21–26) vs 25 (22–28) kg/m^2^; *P* = 0.011). Similar trends were noted for obesity (BMI ≥30 kg/m^2^), but the differences were not significant.

Multiparity and history of ≥2 miscarriages were comparable between both groups.

Prevalence of smoking during pregnancy was higher in TAI+ women vs TAI− (22.7 vs 13.9%; *P* = 0.035).

Table 2 shows thyroid parameters in TAI+ and TAI− women and according to the fetal gender.

**Table 2:** Thyroid parameters in women with and without TAI and according to the fetal gender. Continuous data are expressed as the mean ± SD or as median (IQ 1–3) according to the distribution and categorical data as *n* (%). Statistically significant values are presented in bold.

Demographic and obstetric data	All women	TAI		*P*-level*
		Positive (+)	Negative (−)	
Total, *n* (%)	**1201**	**75 (6.2%)**	**1126 (93.8%)**	
FF	**622 (51.8%)**	**32 (5.1%)**	**590 (94.9%)**	
MF	**579 (48.2%)**	**43 (7.4%)**	**536 (92.6%)**	
Gestational age at blood sampling (weeks)	12 (11–14)	12 (11–15)	12 (10–14)	0.701
FF		12 (11–15)	12 (10–14)	0.555
MF		12 (11–14)	12 (10–14)	0.995
TSH (mIU/L)	1.37 ± 0.75	1.70 ± 0.79	1.35 ± 0.74	**<0.001**
FF		1.64 ± 0.79	1.34 ± 0.70	**0.016**
MF		1.74 ± 0.79	1.36 ± 0.79	**0.003**
FT4 (pmol/L)	13.8 ± 1.70	14.0 ± 1.80	13.8 ± 1.70	0.372
FF		13.6 ± 1.90	13.8 ± 1.70	0.439
MF		14.3 ± 1.60	13.8 ± 1.70	0.067
TPOAb (kIU/L)	28 (28–37)	271 (90–1014)	28 (28–34)	**<0.001**
FF		292 (92–1094)	28 (28–34)	**<0.001**
MF		216 (87–996)	28 (28–34)	**<0.001**

TSH, thyrotropin; FT4, free thyroxine; TPOAb, thyroid peroxidase autoantibodies; TAI, thyroid autoimmunity; FF, female fetus; MF, male fetus; SD, standard deviation.

* TAI+ all vs TAI− all/TAI+ FF vs TAI− FF/TAI+ MF vs TAI− MF; *P* values were considered significant whenever *P* < 0.017 (0.05/3) for three comparisons.

Serum TSH levels were higher in TAI+ vs TAI− women (1.70 ± 0.79 vs 1.35 ± 0.74 mIU/L; *P* < 0.001) even after excluding patients with overt hypothyroidism or SCH. This difference in serum TSH was independent of a pregnancy with a FF or a MF.

FT4 levels were comparable between both groups (14.0 ± 1.80 vs 13.8 ± 1.70 pmol/L; *P* = 0.372) and there were no differences by fetal gender.

Table 3 shows the prevalence of GDM in TAI+ and TAI− women and according to the fetal gender.

**Table 3:** Prevalence of GDM in all women and according to the TSH groups. Continuous data are expressed as the mean ± SD or a median (IQ 1–3) according to the distribution and categorical data as *n* (%). Statistically significant values are presented in bold.

Pregnancy and obstetrical outcomes	All women	TAI		*P*-level*	*P*-adjusted
		Positive (+)	Negative (−)		
Total, *n* (%)	**1201**	**75 (6.2%)**	**1126 (93.8%)**		
FF	**622 (51.8%)**	**32 (5.1%)**	**590 (94.9%)**		
MF	**579 (48.2%)**	**43 (7.4%)**	**536 (92.6%)**		
GDM	235 (19.6%)	21 (28%)	214 (19.0%)	0.057	**0.008**
FF		11 (34.4%)	113 (19.2%)	0.036	**0.002**
MF		10 (23.3%)	101 (18.8%)	0.479	0.458

* TAI+ all vs TAI− all/TAI+ FF vs TAI− FF/TAI+ MF vs TAI− MF; *P* values were considered significant whenever *P* < 0.017 (0.05/3) for three comparisons.

GDM, gestational diabetes mellitus; SD, standard deviation; TAI, thyroid autoimmunity; FF, female fetus; MF, male fetus; N.A., not applicable; adjusted *P* values: for GDM taking into account obesity, age >34 years and previous GDM.

The overall prevalence of GDM was 19.6%. GDM prevalence was higher in TAI+ vs TAI− women (28 vs 19%; *P* = 0.008), but this difference was only significant in TAI+ women pregnant with a FF (34.4 vs 19.2%; *P* = 0.002) and not in women pregnant with a MF (23.3 vs 18.8%; *P* = 0.458). In TAI+ women, the prevalence of GDM tended to be higher in women who were expecting a FF (34.2%) vs a MF (23.3%); *P* = 0.814 and *P* = 0.528 after adjustment for confounders, data not shown in the table.

## Discussion

Our first major study observation was that euthyroid TAI+ women have a higher prevalence of GDM than TAI− women.

A recent original study and a meta-analysis noted an increased risk of GDM in euthyroid TAI+ women (OR: 1.09 and 1.65, respectively) (8, 12). However, other studies did not observe this association (13, 14). In our study, the prevalence of GDM was 28% in women with TAI, and 19% in women without TAI. Several mechanisms have been suggested which might link TAI+ with GDM via increased IR, a key element in GDM (7, 15). One pathway is through a common inflammatory pathway involving factors such as interleukin-6 (IL-6), IL‐12, IL‐10 and tumor necrosis factor that were shown to be significantly higher in women with autoimmune hypothyroidism (15, 16, 17) and euthyroid TAI+ women (16) compared to TAI− controls. Another pathway is through raised TSH, since TAI is the major cause of SCH, and the latter has been associated with higher IR through mechanisms including reduced expression of the GLUT-4 glucose transporter and lower blood flow in adipose and muscle tissue (7, 17). In a meta-analysis, the presence of TAI combined with SCH (TSH levels >4.0 mIU/L) resulted in an increased risk of GDM (OR: 2.04 (95% CI: 1.32–3.13)) (18). We excluded women with SCH in our study, but serum TSH levels were nonetheless significantly higher in TAI+ women vs TAI− women with a mean difference of 0.35 mIU/L.

In a recent study, higher human chorionic gonadotropin (hCG) levels were associated with a lower prevalence of GDM, some of which effects appeared to be related to FT4 levels (19). However, when results were adjusted by TAI+ status, the association between hCG and GDM did not persist. Indeed, in two studies it was shown that the impact of serum hCG on thyroid function during pregnancy was impaired in TAI+ vs TAI− women (both in case of the presence of TPOAb and TgAb) (20, 21). It was hypothesized that lymphocytes may result in impairment via interference with hCG bioactivity.

Thus, the reasons explaining the higher prevalence of GDM in TAI+ women remain unknown, with the potential role of hCG meriting further investigation.

Our second main observation was the higher prevalence of GDM in TAI+ women, but only in pregnancies with a FF. Our study is one of the few to investigate the impact of TAI on pregnancy outcomes according to the fetal gender. The only other study to do so did not find a higher prevalence of GDM in women pregnant with a FF (13). The mechanism of this gender-mediated difference remains speculative.

In studies investigating GDM as outcome, adjustments are made for age, BMI, previous GDM and a history of diabetes in a first-degree relative but are not typically performed for fetal gender (22). In our study, the prevalence of BMI ≥30 kg/m^2^, previous GDM, smoking, non-Caucasian background and multiparty were comparable between TAI+ and TAI− women, and between fetal gender groups. The only (significant) difference was a higher prevalence of age ≥35 years in the TAI+ group (and a trend in TAI+ women pregnant with a MF), but despite that, women pregnant with a MF had a lower prevalence of GDM compared with women pregnant with a FF.

Lower hCG levels are associated with lower stimulation of the thyroid gland, and therefore, lower FT4 and higher prevalence of GDM (19, 23). In the general population, this hypothesis might explain the higher prevalence of GDM in women pregnant with a MF vs a FF, taking into account the lower hCG levels observed in pregnancies with a MF vs a FF, a difference that is observed already soon after conception (3, 4, 5, 24). Furthermore, in a previous study, we showed that women pregnant with a FF had lower TSH levels (25). These differences in thyroid function can be explained by higher hCG levels when pregnant with a FF and/or other pro- and antiangiogenic factors, such as placental growth factor and soluble FMS-like tyrosine kinase-1 (sFit-1), respectively (26). However, the impact of hCG is inadequate if TAI is present due to an impaired action of hCG on the thyroid gland (19). Finally, sex differences in the genetic influences on TSH levels have been described, being more important in females than in males (75 and 41%, respectively) (27, 28). In our study, the prevalence of GDM was higher in women pregnant with a FF, and therefore these hypotheses do not seem to hold up.

In pregnancies with a FF compared with a MF, greater stimulated production of IL-6 have been reported during all trimesters, TNF-α in early pregnancy, and IL-1β in mid- and late pregnancy (29). In the first trimester, a more pro-inflammatory profile (IL-6 and TNF-α) has been reported in women pregnant with a MF, but that changes from the second trimester onward (30). The immune profile in pregnancies with a FF vs a MF is similar to that in women with GDM and characterized by a higher proportion of Th2, Th17 and Treg cells compared with women without GDM (31). Finally, differences in sex hormones and in concentrations of TGF-β (a multifunctional cytokine associated with GDM) between women pregnant with a FF vs a MF could contribute to this association (32, 33, 34).

The association of fetal sex on pregnancy-related and fetal outcomes should be of interest to researchers (35).

Limitations of our study were the absence of measurement of hCG, TgAb status, which is known to be present in around 5% of women without TPOAb and which is associated with an impaired impact of hCG on thyroid function during pregnancy, and other placental factors (21, 36, 37).

## Conclusions

In our study, euthyroid TAI+ women had a higher prevalence of GDM compared to TAI− women, but this difference was only present in pregnancies with a FF. These study results should be confirmed, and the underlying mechanisms studied with the measurement of interleukins, serum hCG and other placental factors.

## Declaration of interest

K G Poppe is secretary of the European Thyroid Association and received lecture fees from the Berlin-Chemie AG, IBSA and Merck Healthcare KGaA. All authors declare that there is no conflict of interest that could be perceived as prejudicing the impartiality of the work reported.

## Funding

No specific grant/fellowship from any funding agency in the public, commercial or not-for-profit sector.

## Author contribution statement

MP and ML drafted the first version of the manuscript. FV collected data and revised the manuscript. PK, GS and LG revised the manuscript. SR revised the manuscript and approved the final version. KGP designed and performed the study, acquired and analyzed the data, revised the manuscript and approved the final version.

## Data availability

Data are available for insight at request of the editor.

## Statement of ethics

The study was performed in accordance with the guidelines of the Declaration of Helsinki. The study was approved by the institutional review board ‘Comité Local d'Éthique Hospitalier, N° d’agréation: O.M. 007 AK/15-11-114/4568’, Centre Hospitalier Universitaire C.H.U. Saint Pierre, Rue Haute 322, 1000 Bruxelles. No written consent was obtained from the participants, since the publication was a retrospective analysis of collected data.
